# Complete Genome Sequences of Six Avian-Like H1N1 Swine Influenza Viruses from Northwestern China

**DOI:** 10.1128/genomeA.00098-12

**Published:** 2013-01-31

**Authors:** Jing-Yu Wang, Juan-Juan Ren, Yuan-Hao Qiu, Hung-Jen Liu

**Affiliations:** a College of Veterinary Medicine, Northwest A&F University, Yangling, China; b Institute of Molecular Biology, National Chung Hsing University, Taichung, Taiwan; c Agricultural Biotechnology Center, National Chung Hsing University, Taichung, Taiwan

## Abstract

Very little is known about swine influenza in northwestern China. Here, we report the complete genomic sequences of six avian-like H1N1 swine influenza viruses (SIVs) isolated in pigs in northwestern China. Phylogenetic analyses of the sequences of eight genomic segments demonstrated that they are avian-like H1N1 SIVs.

## GENOME ANNOUNCEMENT

Three predominant subtypes of swine influenza virus (SIV) circulate worldwide in pigs: classical swine H1N1, avian-like H1N1, and the human-like H3N2. Pigs play an important role in the transmission chain of avian-to-pig-to-human, and SIV can be transmitted to human beings across species (1–3). New SIV strains can cause economic losses in the pig industry and pose serious threats to human health (4, 5). These avian-like H1N1 viruses emerged in Europe in 1979. In China, the classic SIV was first isolated in 1974, while the avian-like H1N1 virus was first detected in 1993 to 1994 and cocirculated with the classic H1N1 SIVs (6). After that, similar viruses were isolated in many provinces (7, 8). To date, the prevalence of SI in northwestern China remains unclear. The Shaanxi province is located in the northwest of China and is the transportation hub of the northwest provinces. Understanding the prevalence and variations of SIVs in this region is greatly significant to the prevention and control of SI in China.

A total of 500 nasal and tracheal swab samples were collected regularly in slaughterhouses and farms from healthy pigs in the Shaanxi province from 2011 to 2012. The hemagglutination inhibition (HI) antibody positive rate of H1N1 SI reached 16.9 to 36.9%. Six avian-like H1N1 SIVs were isolated in the Shaanxi province. Of these isolates, the full-length sequences of eight genomic segments were amplified by reverse transcription and PCR and were sequenced and compared with published sequences. The homology between the eight genomic segments of these isolates was 98.3% to 99.1%. The hemagglutinin (HA) and neuraminidase (NA) sequences of these isolates were closely related to those of avian-like H1N1 SIVs from other provinces in China, but the homology with those from European countries is low. Three amino acid residue substitutions (V151I, D204V, and Y209H) at the receptor binding sites in HA among these isolates may affect their binding to cell receptors. Sequence analyses also indicated that positions 119 (E), 152 (R), 275 (H), and 295 (N) of NA protein were conserved, suggesting that they are still sensitive to neuraminidase inhibitor drugs (9, 10). In addition, a mutation found at position 44 of NA, a glycosylation site, may affect protein folding and transport (11). The amino acid residue at position 375 of PB1, which is the key to the host characteristics (12), is conserved in these isolates. The virulence is strong when the amino acid residue 627 of PB2 is K, and weak when it is E (13, 14). Interestingly, the position 627 of PB2 is E, rather than K, among these isolates. In addition, since an amino acid substitution at position V27I of M2 was seen in 2 of 6 isolates, these viruses seem to have a certain resistance to amantadine drugs (15). Phylogenetic trees based on eight genomic sequences of these isolates suggested that they are closely related to avian-like HIN1 SIV strains from China and Europe.

### Nucleotide sequence accession numbers. 

The GenBank accession numbers of these isolates from Shaanxi province are shown in Table 1.

**TABLE 1:** Nucleotide sequence accession no. of six avian-like H1N1 SIV strains isolated from Shaanxi province of China

Isolate	PB	PB1	PA	HA	NP	NA	M	NS
A/Swine/Shaanxi/s1/2011 (H1N1)	JX860681	JX860680	JX860682	JX860678	JX860683	JX860679	JX860685	JX860684
A/Swine/Shaanxi/sf/2011(H1N1)	JX860697	JX860696	JX860698	JX860694	JX860699	JX860695	JX860700	JX860701
A/Swine/Shaanxi/Sw/2011(H1N1)	JX963601	JX963600	JX963602	JX963598	JX963613	JX963599	JX963603	JX963604
A/Swine/Shaanxi/s2/2012(H1N1)	JX963608	JX963607	JX963609	JX963605	JX963614	JX963606	JX963610	JX963611
A/Swine/Shaanxi/s3/2012(H1N1)	JX963593	JX963592	JX963594	JX963591	JX963595	JX963612	JX963596	JX963597
A/Swine/Shaanxi/S6/2012(H1N1)	JX860689	JX860688	JX860690	JX860686	JX860691	JX860687	JX860692	JX860693
